# Supplementary material for: STARCall integrates image stitching, alignment, and read calling to enable scalable analysis of in situ sequencing data
Source: PLoS Comput Biol. 2026 Apr 27;22(4):e1013689. doi: 10.1371/journal.pcbi.1013689 (PMC13160441; doi:10.1371/journal.pcbi.1013689)
Supplement: Attachment — Submitted filename: Response-to-reviewers.pdf [file pcbi.1013689.s002.pdf]

Response to reviewer comments:

Reviewer #1: The authors present a pipeline for performing read calling of in situ sequencing starting from image sequences of the fluorescently labeled nucleotides. The pipeline's novel algorithms for image alignment algorithm and spot-to-base calling build on existing state-of-the-art methods, claiming improved accuracy and robustness. Image alignment and base calling performance is evaluated on a number of datasets, some provided by the authors and some from other publications. The performance evaluation supports the claim that this pipeline is a marked improvement over existing methods, and the authors identify known weaknesses and areas of potential improvement. An end-to-end snakemake pipeline is provided for ease of use as well as standalone python packages for flexible integration with other analyses. Overall this work is a novel and valuable contribution to the field.

I would recommend this manuscript for publication with minor changes and clarifications as follows:

1. There is no mention of flat field image correction in the pipeline. All microscopes suffer from vignetting to some degree, causing measured fluorescence intensity to drop off by 50% or more at the edges of an image. Given that the text claims a 10% drop in spot intensity due to subpixel image shifting leads to a read miscall rate of 15%, it seems like a 50% drop due to vignetting would be catastrophic. Is your microscope software already applying illumination correction? Or perhaps the base calling algorithm is somehow insensitive to vignetting yet highly sensitive to the subpixel shift issue? This needs to be explained in the text.

In the analysis we performed we did not do any flat field image correction of the images. We found the images obtained from our microscope have a minimal level of vignetting, and opted to not complicate the procedure. We agree that this is a common problem that affects many microscopy images, and to help minimize the effects on analysis steps we now include the BaSiC illumination correction package in STARCall, which can be enabled if needed. However, we don't believe that vignetting would cause as large of a problem as subpixel image shifting, because vignetting affects the intensity of both the spots and background. The 10% drop in spot intensity we measured from subpixel shifting hinders basecalling because only features a few pixels in size are affected, so the signal we want (e.g. the pixel-sized spots) are reduced in intensity whereas the background is not. Because this signal-to-noise ratio is what is damaging to read calling, not the absolute intensity change, we changed our subpixel shift test to measure the ratio of dot intensities to the mean pixel intensity of the image. We feel this better represents the factors driving the increase in miscalling caused by the subpixel shift.

"STARCall can also call tools such as CellPose to segment cells, BaSiC to perform illumination correction, and CellProfiler to compute cell features from the phenotyping images" (lines 113-115)

"Another issue that is common for microscopy images is vignetting; we did not apply illumination correction to remove the effects of uneven illumination from the images before stitching. These uncorrected images yielded accurate base calls. However, our analysis of subpixel shifting revealed that loss of dot intensity relative to background dramatically increased the miscalling rate, raising the possibility that severe uneven illumination might degrade read calling performance. We speculate that STARCall is less sensitive to such uneven illumination because it affects the intensity of both dots and background, unlike subpixel shifting. However, STARCall can call BaSiC to perform illumination correction if desired." (lines 394-402)

“d) Ratio of mean dot intensity to mean pixel intensity (top) and percent of reads miscalled (bottom) when sequencing images are shifted by a fractional pixel offset using spline interpolation.” (Fig 2 legend)

2. Figure 2 f,g,h: The error metric values for MIST and ASHLAR seem quite different from what was reported in their respective papers (even after correcting for different units – microns vs. pixels – in the ASHLAR case). Specifically, I am looking at MIST Table 1 and Ashlar Figure 5A/5B. Can you explain these discrepancies?

We thank the reviewer for highlighting these discrepancies. For the MIST dataset, we found two errors in our code reproducing their analysis, one being that we were measuring the absolute value of the error in area, and the other being that we did not use the calibrated segmentation thresholds included in the supplement. After fixing these errors, our results matched very closely to the values reported in Table 1. These corrections did not change the difference in performance between ConStitch and MIST, as shown in the updated figures (Fig 2f,g).

For the ASHLAR dataset, we did not find any errors in our implementation, however we tested multiple parameter configurations for running ASHLAR, finding a configuration that performed the same as what was reported in the ASHLAR paper. We describe this configuration as well as all commands used to invoke ASHLAR in a new supplemental table (Table S1). With these changes ASHLAR performs better than ConStitch (median alignment error of 0.09 vs. 0.34) on the image set only when interpolation is enabled for ASHLAR. This result aligns with our previous results comparing ASHLAR to ConStitch on the other image sets. We have updated the text and figure legend to reflect this change.

3. Figures overall: A lot of panels are missing appropriate units on some of the axes. Images lack scale bars. (In figures containing multiple same-scaled image fields, you can add the scale bar to just one convenient field)
1A: scale bar
2B: scale bar
2C: Y axis - what is the unit for alignment error?
2D: upper panel Y axis: What is the dot intensity unit? (A.U. is OK, but say so)
2E: X axis
2H: X axis
2J: X axis
3C: Y axis (both panels)

We thank the reviewer for noting these omissions, and have added units and scale bars to all figures except 2B, where cell images are provided only as part of a schematic/cartoon.

4. Lines 61, 110, 194: The text does not explain how alignment of images from different objectives or microscopes are handled. Different objectives or microscopes will have different magnification and/or sensor pixel pitch, leading to images with different nominal image resolution (pixel size) which phase correlation alone cannot handle. The manuscript must explain how this is addressed in the algorithm.

We agree that we did not explain the alignment process for images taken on different microscopes in sufficient detail, given that it is a complex problem to solve and that phase correlation cannot handle differences in resolution. ConStitch has an optional scale_factor parameter, which specifies the relative scale of image tiles which can be used to deal with images taken at different resolutions. Before pairwise alignment of two images, if the scale factors differ, the image with the smaller scale factor is upscaled to match the larger image. The parameter value is usually straightforward to determine, as most microscopes store the pixel size of images as metadata. The main text and methods sections have been updated to include these details.

“If the two images have differing scale factors, e.g. they were taken with different objectives, the image with the smaller pixel size is scaled up to match the resolution of the other image before phase cross correlation is applied. The pixel size of images is typically included in the metadata recorded by the microscope, and is extracted by ConStitch.” (lines 148-152)

5. Line 222: Gaussian filters can be parameterized in multiple ways, and describing it as “1 pixel” is not precise enough. I assume the authors meant “a Gaussian filter with a standard deviation of 1 pixel”. Please clarify in the text.

We appreciate this comment, and have revised our descriptions of Gaussian filters to fully specify the parameters of the filter.

“We next apply a Gaussian filter with standard deviation 1 to reduce any noise introduced by this procedure.” (lines 291-292)

6. Line 289: Here the stitching alignment is claimed to provide “subpixel alignment,” but the algorithm forces image positions to integer pixel coordinates. Please explain this discrepancy.

We use the term “subpixel alignment” because the algorithm used to quantify misalignment returns a fractional amount of alignment error. Using this we referred to <1 pixel of alignment error as “subpixel alignment”. However we agree the meaning of this term is confusing, and we have replaced it with “precise alignment”.

7. Remember to provide all data used in creating the various figure plots, as per the PLOS Data Availability Policy: “For example, authors should submit the following data: The values behind the means, standard deviations and other measures reported; The values used to build graphs; …” The preferred approach is submitting the data files to an accepted public data repository.

We have collected all data used to create figures, including the alignment error of all regions in all image sets, the reads for all cells with their matching barcodes, and the resource usage data. Values behind all summary statistics are included in these tables, which are now hosted on Zenodo at (10.5281/zenodo.19027452).

Reviewer #2: Specific questions/comments:
1. “This resulted in more than 50% of tiles with <1 pixel alignment error on all nine image sets, while ASHLAR had higher error on four image sets” - How big was the difference between the image sets? Is there a pattern in images where ASHLAR and STARCall have similar quality and when they differ?

The difference in percent between ConStitch and ASHLAR ranges between 35% for image set 1.1 and 14% for image set 2.1, with a mean of 25%. The performance of ConStitch and ASHLAR are generally correlated across image sets, with a mostly consistent difference between them. Both ConStitch and ASHLAR perform well on the Feldman image set, which makes sense as that image set was manually aligned to ~10 pixels, meaning much less alignment was needed. In addition they both performed worst on image set 1.2. We looked to see if there were any specific regions of the image set in which ConStitch and ASHLAR both had elevated alignment error, but the alignment error appeared evenly distributed across the image set.

We have revised the sentence in the abstract to be more precise: “Overall, STARCall aligned more than 50% of tiles with <1 pixel residual misalignment on all nine image sets, outperforming alternative packages by 14-35%” and in the introduction to be “This resulted in more than 50% of tiles with <1 pixel residual misalignment on all nine image sets, outperforming ASHLAR by 14-35%” (lines 19-20).

2. Fig 2b – ‘Filter out regions without features’ – what does filter out mean? What if there is useful information in those regions that gets filtered out?

By “filtered out” we mean that estimated alignment for the region is not considered when finding global positions for each tile. We did this because, if a region is filtered out, it has a low correlation score and therefore has limited to no features that can help with alignment. However, regions that are not used to globally position tiles are still included in the stitched image so no information is lost. We have amended the figure legend to clarify this point.

“b) Procedure for stitching and aligning multi-cycle image sets. Overlapping pairs of images are aligned to each other, regions with no features are excluded from the alignment process, then the system of equations containing all pairwise alignments is solved to find global positions for each image.” (Fig 2 legend)

3. Fig 2c. caption ‘Gaussian noise of sigma=100 was added’ – what is the sigma for the image prior to adding gaussian noise? What dtype is used?

The noise was generated by calling random.Generator.normal(scale=100), which returned floating point values, which were clipped to be nonnegative, then cast to the dtype uint16 and added to the images.

4. Fig 2f. median percent error of ROI area is higher for Constitch as opposed to MIST – is this an acceptable trade-off?

During revisions, we corrected errors in our method for scoring the MIST test set (see response to Reviewer 1, comment #2). In doing this, the error of ROI area distributions are now nearly identical, with ConStitch providing a median error of -0.2 while MIST provides a median error of -1.3. Updated histograms of the distribution of error are shown in Fig 3b.

5. In the text figure 2b step 2, etc are mentioned. It would be good to add an annotation withing the figures itself for step number for clarity.

We updated the figure to include labels on each step, we agree that this improves readability of the references to the figure.

6. "A linear model is trained on the remaining constraints to estimate physical parameters of the microscope such as the travel between tiles and the camera angle" – this is interesting – how well does a linear model fare and are there similarities observed between microscopes?

We found that the linear model did a good job learning the physical parameters of the microscope. The MIST image set (Fig 1f,g) provides a good test of the linear model, as it contains sparse stem cell colonies that can only be stitched together with the linear model filling in constraints for regions that have no features. Here, out of the 1,057 image pairs that were overlapping, only 228 had sufficient features to be aligned, and the rest had to be estimated using the linear model. ConStitch performs nearly as well as MIST in terms of positional error, which suggests that the linear model successfully learned the parameters of the microscope.

7. MAE solver is likely more robust because it is less affected by outliers as compared to MSE, which penalizes large deviations heavily.

We agree with this point, we find a large benefit of the MAE solver is that it is more resistant to outliers, and thus the filtering step does not have to be as precise. We added this point to the main text, highlighting the benefit of using MAE over MSE:

"The Spanning tree, MSE solver, and MAE solver methods had similar performance on images that align well, but the MAE solver performed better in non-optimal imaging conditions. We believe this is due to the MAE solver being robust to outliers, which enables it to handle erroneous constraints that are still present after filtering." (lines 173-176)

8. The result from interpolation vs 1 pixel alignment error is interesting. What type of interpolation was used?

Spline interpolation was used, the same implementation used by ASHLAR, specifically the implementation in the scipy library at scipy.ndimage.shift. We agree the results are interesting, and we

believe that interpolation is damaging to read calling because single-pixel features will be spread out over multiple pixels in the interpolated image, resulting in lower dot intensities.

9. Removing unwanted high frequency features such as cell debris using subtraction method is simple and effective. However, if the high frequency feature has different intensity across images they might still disrupt the algorithms, even if the subtracted intensity is low. How likely are such variations to occur? Is the standard deviation always low in such cases?

We agree that it is possible for unwanted features to remain after the subtraction filtering step. However, based on manual inspection of the images we found that most background or noise that is bright enough to overwhelm the amplicon colonies after subtraction does not change in intensity between cycles. The type of noise that typically changes between cycles is the background, which is quite diffuse and not high frequency.

10. STARCall CPU time is significantly higher than Feldman et al. Are there any plans for leveraging GPUs for speeding up computation?

Currently STARCall is equipped to use GPUs for the cell segmentation step, as the libraries it uses support GPU inference of the deep learning models used for segmentation. This was not enabled when measuring the runtime of STARCall, but would provide a speedup if used. Other than this step, there are no plans to add GPU execution.

We updated the section on resource utilization to include “We did not utilize GPU execution to speed up the cell segmentation step, but the underlying libraries Cellpose and Stardist both support GPU execution. STARCall provides a configuration option to enable GPU execution, which would reduce cell segmentation execution time.”

11. How was the diameter parameter for Cellpose segmentation chosen?

The diameter parameter was chosen through manual inspection of the phenotype images, and by manually measuring the size of a small sample of cells. Segmentations were inspected for each image set to ensure performance was acceptable.

Reviewer #3: This paper presents STARCall, a computational pipeline for stitching, cycle-to-cycle registration, and read calling of in situ sequencing image datasets which additionally interfaces with existing open source cell segmentation and cell image phenotyping packages (Cellpose, Stardist, and CellProfiler).

**** Summary ****

--- stitching ----
(A1) Relative translations for all tile overlap regions are estimated using Fourier Phase Correlation (FPC).
(A2) Overlaps with insufficient image features for registration are automatically detected and removed if their post FPC score is below the 95th percentile of scores for FPC alignments of randomly selected non-overlapping regions.
(A3) A linear model is fit to the retained translations which provides translations for overlaps removed in the previous step; essentially, missing translations are linearly interpolated from known translations.
(A4) Each tile has an optimal translation with respect to each of its neighbors - which do we accept? This over constrained system is solved for single optimal positions for each tile with an integer linear program (ILP) that minimizes the sum of "slack variables." The "slack variables" are defined as the absolute error between a tiles position and the neighbor-optimal translation found with FPC.

--- cycle-to-cycle alignment ---
(B1) This crucial step is not thoroughly described anywhere in the main text or methods section. This is an essential missing detail that should be described in explicit detail before this paper is considered for publication.

--- read calling ---
Aligned data can be considered a 4-dimensional array: f(t, c, x, y) for cycles, channels, width, and height.
(C1) For all t and c, a Gaussian smoothing of sigma=3 pixels defines a low frequency background which is subtracted from the original images.
(C2) To isolate signal amplitude of true positive labels, the second maximum over c is subtracted in a pixel wise manner at all locations x and y and for all cycles t.
(C3) For all t and c, a Gaussian smoothing of sigma=1 pixels is applied to remove high-frequency noise.
(C4) To highlight amplicon colonies, the standard deviation is taken over t then a sum is taken over c.
(C5) A Laplacian of Gaussian (LoG) filter detects local maximums in the 2D image from the previous step.
(C6) A base pair read is called at every LoG detected point as the maximum value over c at that spatial location.

--- experiments ---
(D1) A few implementation details are compared: the ILP solver and interpolation method used when spatially shifting tiles/cycles.
(D2) The MIST dataset includes evaluation images wherein cell colonies are carefully centered in the single tile acquisition field of view. A second acquisition of the same sample is arbitrarily tiled. Ideally, after the second dataset is stitched, cell colony positions and areas (obtained by post-stitching segmentation) should not significantly differ from the evaluation images.
(D3) This paper asserts that after stitching and cycle-to-cycle alignment any residual misalignment in image sub-regions must be induced by stitching error (rather than true sample deformation between cycles). 200x200 pixel sub-regions from stitched images are registered with FPC between cycles; the measured shift is considered stitching error. When the authors frequently state "less than 1 pixel alignment error" it is with respect to this indirect measure that they are referring.

(D4) The percent of segmented cells which are confidently genotyped after read calling is provided as a measure of success with no effort to quantify false positive or false negative rates.
(D5) Given that the target sequences are known, the frequency of bases in the library sequences should approximately match the frequency of bases called by the read method. This comparison is also provided.

--- implementation, software, and performance ---
(E1) All software is open source and at first shallow glance the repositories seem well documented and well organized.
(E2) Run times using a 100 CPU + 1TB RAM cluster job, as quantified by the cluster manager itself (from the appearance of the graphs) are presented.

**** Comments ****

--- stitching ---
Translation only stitching using FPC is a very well established method, nothing in steps A1, A2, or A3 appears novel to me. Regarding step A4, given the slack variables definition, it seems to me that after accounting for cancelations and doing some algebra the ILP objective function reduces to exactly the cumulative error between tile positions and the overlap constraints and is thus only trivially different from what is used in reference 18 by using MAE instead of MSE. Scaling the constraint equations by the overlap FPC scores seems novel, but also dubious. For realistic image data, in my experience correlation is a useful relative measure, but not a useful absolute one. The correlation of a well aligned overlap region can be significantly changed by adding noise or removing/adding a few cell colonies. So, the FPC score is as much a measurement of the content contained in the overlap region as it is of the alignment quality. Overall I would say the method used here is a standard and well established one with a few tweaks cast to an ILP formulation.

We thank the reviewer for these comments, and have removed claims of novelty regarding these steps. Instead, we highlight the utility of combining these steps into a new and useful pipeline.

--- cycle-to-cycle alignment ---
A thorough description of this is not apparent in the text anywhere that I can find. The methods section on stitching and alignment should be expanded to discuss alignment across the cycle axis in as much detail as alignment across tiles is described; especially because the authors claim that doing stitching and cycle-to-cycle alignment jointly improves upon prior approaches. If the authors want to keep this claim in the paper, it should be directly supported by experiments that compare a joint optimization to separate optimization of tile and cycle alignments.

We thank the reviewer for this comment, we agree that our explanation of cycle-to-cycle alignment was not sufficient given that it is the main improvement proposed in ConStitch. Cycle-to-cycle alignment is accomplished by ConStitch by including all cycles in one single adjacency graph, and adding edges between tiles that are in the same position but in different tiles. The pairwise alignment procedure used to refine the alignment of two overlapping tiles is applied to all edges, both intra-cycle (between two neighboring tiles in the same cycle) edges and inter-cycle (between two tiles in the same position but different cycles) edges. Using an adjacency graph containing all cycles allows us to perform joint optimization of both stitching and alignment. We have updated the results text to clearly describe this procedure for cycle-to-cycle alignment:

“Like MIST and ASHLAR, ConStitch operates on an adjacency graph of all images, where each image corresponds to a node and edges connect all pairs of overlapping images. ConStitch extended this approach to accomplish cycle-to-cycle alignment by including images from all cycles in a single adjacency graph. Two types of edges are added: intra-cycle edges connect adjacent tiles that are in the same cycle, and inter-cycle edges connect tiles in the same position but in different cycles. Thus an adjacency graph containing all cycles is constructed, which allows for joint optimization of stitching and alignment. The adjacency graph is constructed using positional information from the microscope, or a user-defined scanning pattern” (lines 134-142)

In addition we have expanded the methods section to describe this procedure for cycle-to-cycle alignment in detail.

We also agree that we never fully supported the claim that specifically the joint optimization of stitching and alignment is beneficial for alignment. To test this we added an additional solving method to experiment D1 that does not perform joint optimization. Instead, each cycle is stitched independently, then a single translational alignment is found to align each cycle to another. This method resulted in a much higher alignment error (67.2 pixels) compared to the other methods using joint optimization (all <4px), which we believe supports the claim that joint optimization is a vital step in our algorithm (Fig. 2c).

The most basic assumption is that after stitching FPC is again used to find a single global translation from each cycle to a single target cycle. I have never worked with 2D in situ sequencing data, but in my experience with 3D in situ transcriptomics, between cycle registration has always required more than a single global translation. In fact, for the most sensitive experiments, non-linear deformation between cycles has been required. This is highly sample preparation dependent of course, so I'm not doubtful that the authors were careful and accurate in their own experiments, but the idea that this assumed approach could generalize to 3D or more complex samples is very naive.

We agree with the reviewer that non-linear deformation is almost always necessary to align multiple cycles of imaging. After expanding our description on cycle-to-cycle alignment in the results and methods section, we hope that our procedure is now clear. That being said, we have not tested ConStitch on any 3D or complex image sets, and so we agree that the claim that it would be able to generalize to these imaging types is ambitious. We have added a sentence highlighting the difficulties that would come up when applying these algorithms to different imaging types.

“For example, the stitching algorithms in ConStitch can be used in any imaging experiment where multiple rounds of imaging need to be aligned together, including IBEX20 and cyclic immunofluorescence19, with the possibility of being extended to 3D imaging or time series image sets as well. However these imaging methods pose difficult challenges such as nonlinear sample deformation, so application of STARCall may require large changes.” (lines 404-409)

--- read calling ---
Similar to the stitching approach, none of the steps used here on their own seem novel to me. However, I do think they are good choices arranged in a good order and they are similar to other published

approaches for detecting individual bright spots close in size to the image sampling rate (1 - 3 pixels across).

Thank you - we are proud of this work!

I noticed the main text and methods sections describing read calling are not entirely consistent with each other. For example, the main text says a difference of Gaussian filter is used, and the methods section does show both low and high pass filters, but with a critical step in between them, which is not exactly a difference of Gaussians filter. Maybe more importantly, the main text describes z-scoring the channels but the methods section does not. These inconsistencies should be ironed out. The method which was actually used to process data presented in the figures should be described and nothing else.

We thank the reviewer for highlighting these discrepancies between the main text and methods. We have updated both to be clear and consistent in the steps used for read calling.

"To ensure accurate base calling, we remove this background by subtracting a Gaussian blur of the image, applied independently for each cycle and channel." (lines 281-282)

"We then z-score the pixel intensities of each channel and cycle independently." (lines 542-543)

About the z-scoring, it does make sense to do this because the channels are arithmetically combined (a subtraction first, then a sum later). But the authors also make a good point that this assumes similar distributions of bright dots vs. background in all channels. Just a suggestion, an alternative approach would be to do a weak initial spot detection (e.g. just run LoG directly with a low num_sigma), then take the average of the, say, 100 brightest spots and 100 darkest spots and linearly transform the image such that these two numbers equal standard values consistent between channels.

This is a great idea to try, and we like that this approach does not rely on all channels having the same distribution of background and dots. Unfortunately, we did not have time to implement and test this approach. We will keep this in mind as a future improvement for the software.

--- experiments ---
Experiments D1, D2, and D5 all seem fine to me, in particular I find it very interesting that interpolation affected spot reading far more than small spatial shifts.

Personally, I do not think experiment D3 is sufficient to make the claim "less than 1 pixel alignment accuracy." In the image registration literature that kind of claim is usually made in reference to gold standard correspondences, e.g. manual annotations or sparse sets of cells expressing a distinct marker not used for registration itself (an independent validation channel). Experiment D3 uses residual misalignment of sub-regions between cycles instead, which is pretty indirect and confounded by the possibility of real sample deformation.

We agree with the reviewer that our error metric is not directly measuring the alignment error of the stitched image. To better represent this we now refer to the metric as "residual misalignment", and expanded the description of the metric to include disclaimers about its meaning.

"We measured alignment performance by dividing the stitched images into 200 by 200 pixel regions, then applying phase cross correlation to each region to find the misalignment between cycles. This metric does not quantify misalignment relative to any ground truth, instead only measuring the consistency of the alignment against itself. Nonetheless, this metric is still valuable as we already confirmed ConStitch provides comparable stitching performance to MIST and ASHLAR, and we showed how small misalignments can be detrimental to read calling (Fig 2e,f,g)" (lines 218-224)

"This resulted in more than 50% of tiles with <1 pixel residual misalignment on all nine image sets, outperforming ASHLAR by 14-35%" (lines 106-108)

In addition, we performed a new experiment measuring alignment performance using the MIST image set. The MIST image set contains multiple imaging rounds taken with different amounts of overlap between images, which we used as multiple cycles to test alignment performance, with the benefit being that all colonies in the MIST have a ground truth position. The results of this new experiment can be found in Fig 1i,j. This experiment does not support the claim that ConStitch achieves less than 1 pixel of alignment error, but we feel it further supports our claim that ConStitch provides improved alignment compared to ASHLAR.

I also do not particularly like the readout for experiments D4, as it's not really a measure of correctly read cells, just the total number of read cells. One could get 100% on this measure by just assigning a random sequence from the library to every cell. I think taking the time to manually inspect some of the amplicon colonies across cycles/channels and having a manually annotated gold standard set, even for just 100 cells say, would be a more trustworthy measure.

We agree that this experiment does not directly measure the number of barcodes correctly read. Therefore, we manually annotated 500 cells as a test set, and we now also use these manually annotated cells to assess performance. The results of this new analysis can be found in Fig 3e. We believe the addition of this metric supports our claims more strongly and directly than the previous readout.

--- implementation, software, and performance ---
I did not install or run the software myself, but the repositories look welcoming to me. The paper talks about requiring cluster resources. From personal experience every cluster is quite different and moving code from one cluster to another can often require source code changes. Information in the paper on which cluster managers are already supported and how much effort might be required to move the code to a different cluster manager (from slurm to sge for example) would be very helpful.

This is a great point, it can often be quite difficult to port code between cluster environments. However because we use SnakeMake to specify the pipeline, one should be able to avoid some of these code changes. For example, SnakeMake has built in support of Slurm clusters (just requiring the --slurm command line flag), so running STARCall on a Slurm cluster should work with no code changes. Other cluster environments may need larger changes, but all cluster specific configuration has been kept to a single bash script in the repository (run.sh), so changes should be limited to a single file. We have added more information on running the pipeline on different clusters to the readme for the repository on GitHub,

and in the future may be able to provide specific run.sh scripts for different clusters. We have also added a discussion of this point to the manuscript:

“As shown in these tests, the improvements in performance that STARCall provides comes with large resource requirements, larger than alternative methods such as ASHLAR and the Feldman et al. pipeline. These resource requirements make execution on a typical desktop or laptop computer challenging, and all the experiments done here were performed in a cluster environment providing the needed resources. Porting code between cluster environments can be difficult as each cluster is configured differently. However, we implemented STARCall using SnakeMake, which supports many cluster environments including Slurm and common cloud providers. For cluster environments that are not supported by SnakeMake, STARCall centralizes all cluster-specific configuration information in one file that can be easily modified.” (lines 365-374)

I noticed that the cellpose segmentation step took a very long time, something like 8 hours. The paper never mentions a GPU of any kind, so I assume cellpose was run serially on blocks using all cpus to process one block at a time. This is probably not the most efficient way to do this. First, using a GPU is orders of magnitude faster, and second, running multiple blocks in parallel would probably utilize the resources more completely (multiple GPUs would be the best if you have access). I believe cellpose has a distributed version which can handle running blocks in parallel, google "distributed cellpose big data" and you should find it.

Cell segmentation is a time consuming step of the pipeline, and it is true that we ran CellPose only on the CPU which is not ideal. However we are already running multiple blocks in parallel. We divide each stitched and aligned image set into tiles and run each tile through CellPose on its own core. We did not run CellPose on the GPU as we did not have enough GPUs to see a large improvement in processing time compared to distributed CPU execution, however CellPose is fully configured to be able to use the GPU if available. We updated the section on resource utilization:

“We did not utilize GPU execution to speed up the cell segmentation step, but the underlying libraries Cellpose and Stardist both support GPU execution. STARCall provides a configuration option to enable this, greatly improving execution time of cell segmentation given sufficient GPU resources.” (lines 351

**** General comments ****

While I don't think any of the individual steps in this pipeline are by themselves novel, and some descriptions are just missing (cycle-to-cycle alignment), I think the composition of these functions into a single software package targeting an important data type is a worthy accomplishment that should be published. The language in the paper should be temperate about algorithmic novelty and also about criticizing the capability of existing solutions. Regarding that second point, the claim that images taken with different objectives or from different microscopes just can't be registered is made several times in the paper. This is just not at all true. The image registration community has been registering data taken on different instruments for 50 years. Mutual information exists as a registration optimization function for this very reason. Truly novel deep learning methods are being formulated to address this problem as well (e.g. google: Guiding Registration with Emergent Similarity from Pre-Trained Diffusion Models).

We thank the reviewer for their assessment, and we apologize for misinterpreting the state of the field with regards to image registration. We have removed the claims that images from different microscopes cannot be registered with existing methods, and when necessary clarified that we are referring to a specific method and not the field as a whole. We have also softened our claims of algorithmic novelty, highlighting that the novelty is in the entire pipeline, not the individual algorithms.

In particular, the beginning of paragraph 4 of the introduction has been updated to read: "The stitching and alignment requirements for in situ sequencing image sets, including alignment of images collected with different objectives, near perfect alignment between cycles, and stitching of regions with sparse features, are solvable with current stitching and alignment algorithms. However, the number of requirements means that most existing implementations cannot perform all tasks needed. For example, ASHLAR is the current state-of-the-art package for cycle-to-cycle alignment, but it does not support alignment between images collected with different objectives."

Finally, the most important things for me to see this paper get published are:
** Complete description of cycle-to-cycle alignment in main text and methods*
** Soften claims about algorithmic novelty, the novelty is in the pipeline, not really the individual steps*
** Soften claims about performance when the evaluation method doesn't really support it (experiments D3 and D4)*
** A few more comments about what environments are supported by the packages by default (e.g. cluster manager, operating system) and how much coding would be required to run them in a different environment*

As we detail above, we believe we have addressed these concerns with a combination of text changes and new analyses.

Things that would be nice:
** pseudocode descriptions of the pipeline steps for quick reference*

Unfortunately we did not have the time to add detailed pseudocode descriptions of the different steps of the pipeline. However we hope that the improvements to the methods section provide sufficient details for someone looking to understand or implement these algorithms.

Oh and one final suggestion:
At the very end of the pipeline, if a read sequence has more than one match in the library with equal Hamming distance, you discard that read as ambiguous. If, during the read calling, instead of calling each base as the single maximum over all channels, you just assign a probability to each of the four bases based on relative signal across the channels, and you carry this through to the library look up step, you can probably resolve these ambiguous cases. What I mean is, not all base substitution errors are equal if you reference back to the image data. Suppose you have a read sequence with two matches in the library with a Hamming distance of 1. Maybe in one case it's A --> G and in the other case it's an A --> C. Maybe one of these errors is worse than the other with respect to the original image data. If you retain probabilities for each base at each position you can work this out at library look up time. But if you just discard that information and keep the maximum, these errors look the same.

This is a great suggestion, and we agree that this would help improve accuracy of barcode matching. Unfortunately we did not have time to implement and test this against the current method, but we will look into implementing it in the future. We added this to the discussion as a future improvement.
